# Optimization of Real-Time EEG Artifact Removal and Emotion Estimation for Human-Robot Interaction Applications

**DOI:** 10.3389/fncom.2019.00080

**Published:** 2019-11-26

**Authors:** Mikel Val-Calvo, José R. Álvarez-Sánchez, Jose M. Ferrández-Vicente, Eduardo Fernández

**Affiliations:** ^1^Departamento Electrónica, Tecnología de Computadoras y Proyectos, Universidad Politécnica de Cartagena, Cartagena, Spain; ^2^Departamento de Inteligencia Artificial, UNED, Madrid, Spain; ^3^CIBER-BBN, Madrid, Spain; ^4^Instituto de Bioingeniería, Universidad Miguel Hernández, Alicante, Spain

**Keywords:** real-time, EEG, artifact removal, emotion estimation, HRI

## Abstract

Affective human-robot interaction requires lightweight software and cheap wearable devices that could further this field. However, the estimation of emotions in real-time poses a problem that has not yet been optimized. An optimization is proposed for the emotion estimation methodology including artifact removal, feature extraction, feature smoothing, and brain pattern classification. The challenge of filtering artifacts and extracting features, while reducing processing time and maintaining high accuracy results, is attempted in this work. First, two different approaches for real-time electro-oculographic artifact removal techniques are tested and compared in terms of loss of information and processing time. Second, an emotion estimation methodology is proposed based on a set of stable and meaningful features, a carefully chosen set of electrodes, and the smoothing of the feature space. The methodology has proved to perform on real-time constraints while maintaining high accuracy on emotion estimation on the SEED database, both under subject dependent and subject independent paradigms, to test the methodology on a discrete emotional model with three affective states.

## 1. Introduction

The use of Electroencephalography (EEG) signals for emotion estimation has been in the point of view of the field for the last decades. The future use of systems that could perform real-time emotion estimations in subjects under different health conditions would improve the application of therapies in different scenarios. One of the most promising fields for the application of such methodologies is affective human-robot interaction (HRI).

Under the paradigm of emotion recognition, robots will allow the development of automatic systems for the treatment and evaluation of the brain patterns of patients, taking into account the emotional content and, furthermore, to have the ability to adapt their behavior as the mood of the patient changes dynamically.

From the perspective of the field of robotics, emotions estimation can be performed by evaluating the dynamical changes over facial expressions, body language, voice tone, EEG patterns, and physiological signals, related to the equilibrium between the parasympathetic and sympathetic autonomous systems. The EEG is a non-invasive method of high temporal resolution that could allow real-time recognition of emotional responses. Also, it can provide a better understanding of the user's behavior and emotional responses which involve facial expression, tone of voice, or body gestures, which may remain hidden as is the case for patients with expression and mobility problems. Therefore, in this article, EEG patterns will be analyzed and related to emotional responses, as they may provide a different perspective on patients' emotional responses.

Most research studies using EEG have presented methodologies that used offline and supervised artifact removal obtaining high accuracy results, however, often involving the use of complex deep learning machines that require hyper-parameter tuning (Khosrowabadi et al., 2014; Zheng and Lu, 2015; Zheng et al., 2017; Song et al., 2018). Both processes could take up to several days or even weeks of preparation which are not affordable for domains of study where real-time constraints are involved. On the other hand, the problem of real-time recognition has been already addressed by Liu et al. (2010, 2017) using the IADS database and own-produced video database, respectively, using Fractal Dimensions as the main feature for emotion recognition.

As EEG emotion estimation has proved to be affordable in different ways, the next barrier is to perform such task under real-time constraints. This process faces two main problems: online artifact removal and classification with high accuracy results. The former is usually performed in two steps. Firstly decomposing the signals using independent component analysis (ICA) and recompose the signals for the next step. Secondly, visualizing the signals to manually remove the parts which are related to artifacts. The latter involves the following procedures:

- Feature extraction, to represent the information as a set of features.
- Feature smoothing, to remove variability over time.
- Scaling the training samples taking into account the underlying data distribution.
- Dimensional reduction by means of feature selection techniques.
- Model selection and hyper parameterization for optimal generalization.

Finally, the development of such a methodology that could work under real-time constraints must deal with two main obstacles: artifact removal and accurate classification across sessions and subjects.

### 1.1. Online Artifact Removal

The most common artifacts presented in EEG signals are electro-oculographic (EOG) artifacts, muscle artifacts, and 50 Hz background noise. Artifact removal is necessary, as it reduces possible classification errors and reduces the amount of processed information. On the other hand, care must be taken while carrying out such a process, since valuable information in the signals could be damaged.

Taking into account these assumptions, an automatic artifact removal method can be developed using the following approach. Firstly, 50 Hz background noise can be easily removed by a notch filter based on IIR filters. Secondly, EOG artifacts, such as blinking, are often presented within slow frequency bands, below 5 Hz (Rani and Mansor, 2009), while muscle artifacts are usually presented within medium to high-frequency bands 20–300 Hz (Muthukumaraswamy, 2013). Therefore, muscle artifacts are partially removed outside the range of 1–50 Hz when filtering the signals, since this range includes the best frequency bands for emotion estimation: delta (1–4 Hz), theta (4–8 Hz), alpha (8–16 Hz), beta (16–30 Hz), and gamma (30–50 Hz). As several studies report (Zheng and Lu, 2015; Zheng et al., 2015, 2017), the most effective band ranges for emotion estimation, are beta and gamma bands. Finally, EOG artifacts can be effectively removed with real-time constraints by using independent component analysis (ICA) methods combined with wavelet analysis. Although several real-time EOG artifact removal methods have been developed, only two methodologies (Mahajan and Morshed, 2014; Mammone and Morabito, 2014) based on these approaches were tested.

### 1.2. Emotion Estimation

EEG emotion estimation is considered a challenging task due to different factors. Self-evaluation is needed as there is no basic truth about emotion classification and thus, the assessment performed over experienced emotions is a subjective task. Therefore, a series of emotional models developed in the field of psychology must be used to guide the self-evaluation process. The most used, in the field of EEG emotion estimation, are the discrete and dimensional models (Russell, 1980; Roseman et al., 1990). The former is based on the assumption that emotions produce differentiated and independent emotional responses. The latter assumes that emotions are manifested dynamically with subtle inter-relations among them. The use of discrete labels for emotions is based on the affective-defensive emotional model following Davidson and Fox (1982) and Davidson (1992). There is still no clear evidence of whether emotions affect the brain patterns across specific regions or spread over cortical and sub-cortical areas (Kragel and LaBar, 2016). Due to the variability in brain patterns, it is difficult to find invariant patterns across sessions and subjects. In this paper, in total eight electrodes were used, six temporal electrodes and two prefrontal (AF3, T7, TP7, P7, AF4, T8, TP8, P8), since they have proved to be the best brain areas for emotion estimation (Zheng and Lu, 2015; Zheng et al., 2017).

EEG signals can be approached through several domains: time, frequency, time-frequency, information theory, and signal complexity. Features should be stable across sessions to avoid the greatest amount of variability while carrying as much valuable information as possible. To work in that direction, feature smoothing is an effective technique that helps to erase such variability over time. Linear Dynamic Systems (LDS), moving average or Savitzky-Golay (SG) among other techniques can be used (Zheng and Lu, 2015; Zheng et al., 2015, 2017). Regarding scaling, outliers must be taken into account, by choosing an appropriate methodology, since some methods such as standardizing or min-max scale approaches can damage the feature space for the classification step.

One key step in such machine learning strategies is the dimensional reduction stage for the selection of relevant and stable features over time, which faces two main problems: First, in real scenarios there is no access to the underlying distribution related to the target, this makes it difficult to find relevant features in a way that is closely related to bias-variance trade-off (Kohavi and John, 1997). Second, finding the optimal set of features often involves NP-hard search spaces and the selected model must take into account time constraints in real-time scenarios.

Moreover, in the EEG emotion estimation, time series corresponding to trials are split into a series of samples. The main assumption is that time series related to trials are independent of each other but related to the evoked emotion, so the time series is homogeneous within the trial and heterogeneous between trials (Tashman, 2000). This makes the EEG time series be a special case. While in the time series prediction paradigms, as is the case for regression models, past is used to predict the future, supervised learning models assume independence of samples and do not care about the time order of the samples. Therefore, predefined cross-validation schemes for supervised learning algorithms are not suitable for model performance evaluations.

For the dimensionality reduction step, different approaches differ in the way they exploit the relation between features and target (Kohavi and John, 1997). In general, they are defined as filter-based, wrapper-based and embedded methods. On one hand, filter-based methods perform feature selection independently from the learning process and are based on the assumption that a feature that has higher variance may contain more useful information. On the other hand, wrapper-based methods combine feature selection and the learning process to select an optimal feature subset. This is also the case for embedded methods, which perform a penalty against complexity during the learning process to reduce the degree of overfitting or variance of a model by adding more bias.

Wrapper and embedded methods involve the use of nested cross-validation procedures which may lead to increased computational cost and possible overfitting, especially when a small number of observations are available. Also, as mentioned earlier, these processes, when applied with predefined algorithms, do not take into account the particularities of the EEG time series, so that the feature subset estimates are further biased.

Regarding the classifier to be chosen for the methodology, in recent years, very powerful deep learning approaches have been developed and tested in the emotion estimation field (Tripathi et al., 2017; Yin et al., 2017; Song et al., 2018). Although they have proved to be promising tools, they usually require a very large amount of time for hyper-parameter tuning, so there exists a need to find an approach that could yield automatically and in a short time, while still achieving high accuracy performances.

### 1.3. State of the Art

In this work, the proposed methodology is compared with the latest performances in the field of emotion estimation. A set of research studies are used for comparison as they show the best results in terms of accuracy of the results, albeit the experimental conditions which are not completely equivalent due to the real-time constraints imposed on the present study.

First, Khosrowabadi et al. (2014) developed a Biologically Inspired Feedforward Neural Network called ERNN. They produced a database containing 57 subjects using emotionally tagged audio-visual stimuli, achieving an average performance of 70.83 % for arousal and 71.43 % for valence dimensional spaces, using the 5-fold cross-validation method.

Second, Zheng and Lu (2015) produced their own database, SEED, for the estimation of three affective states. Deep Belief Neural Networks (DBNs) were used to analyzing critical frequency bands and channels through the weight distributions of the trained DBNs. With a selection of 12 channels, the best accuracy result obtained was a mean accuracy of 86.65 % using the first 9 trials as the training set and remaining 6 ones as the testing set, Inter-trial (IT), for each subject.

Third, Zheng et al. (2017) explored a set of popular features used in the domain of EEG emotion estimation. The SEED database was used. Differential entropy and together with the Graph regularized Extreme Learning Machine (GELM) classifier outperformed state of the art results. Mean accuracy of 60.93 % was obtained using the Leave-one-out validation scheme (LOO). For the inter-sessions validation scheme (IS) a mean accuracy of 79.28% was obtained.

Fourth, Tripathi et al. (2017) compared the use of both Deep and Convolutional neural networks (DNN, CNN) using the DEAP database. The valence and arousal dimensions were split into three categories. The DNN model achieved 58.44 and 55.70 %, while the CNN model achieved 66.79 and 57.58 %, respectively using the Leave-one-out validation scheme.

Later, Song et al. (2018) developed a novel Dynamical Graph Convolutional Neural Network (DGCNN) tested over the SEED database. Differential entropy features of five frequency bands were combined resulting in average recognition accuracy of 90.40 % using the first 9 trials as the training set and the remaining 6 as the testing set.

Finally, a comparison is made taking into account those experiments where real-time constraints were faced. As mentioned above, Liu et al. (2017) developed a real-time emotion recognition system which uses a three-level classification approach and a real-time artifact removal algorithm. Regarding the classifying strategy, in the first level, high-arousal and valence emotions versus neutral emotions were estimated with an average accuracy of 92.26. For the second level, positive vs. negative emotions, with an average accuracy of 86.63 were estimated. For the last level, joy, amusement, and tenderness were classified at an average accuracy of 86.43. The training and validation scheme was done by using 8 trials to elicit 7 discrete emotions and one neutral state and the same amount of stimuli as a test set in a real-time emotion estimation scenario.

## 2. Methodology

The objective of the present paper is to perform the whole process involved in EEG emotion estimation under real-time constraints. To prove its feasibility, it will be tested on the SEED database. The process comprises six main steps:

1. Online artifact removal: EAWICA.
2. Feature extraction: Differential Entropy, Amplitude Envelope, Petrosian Fractal Dimension, Higuchi Fractal Dimension, and Fisher Info.
3. Feature smoothing: Savitzky-Golay filter.
4. Feature scaling: Quantile transform followed by min-max scaler.
5. Feature selection: Based on the chi-squared statistic.
6. Classification: Nearest Neighbors (KNN), Support Vector Machines (SVM) with linear and radial basis function kernels, decision trees, random forest, AdaBoost, naive Bayes, and Quadratic Discriminant Analysis (QDA).

### 2.1. SEED Database

The SEED database (Zheng and Lu, 2015) has 15 subjects but the experiment was performed three times each, with a time interval of one week. Emotions were quantified in terms of three discrete categories: POSITIVE, NEGATIVE, and NEUTRAL. A set of 15 emotional-tagged videos were employed, each approximately 180 s long. The international 10–20 system for EEG acquisition was used with a set of 62 channels.

### 2.2. Online Artifact Removal

Two main approaches that are affordable in real-time constraints are used: EAWICA (Mammone and Morabito, 2014), and ICA-W (Mahajan and Morshed, 2014) methods. The performance over artificial artifactual data is first analyzed to compare them under controlled conditions but finally, are compared over real EEG samples obtained from the objective SEED database.

Both approaches use the same underlying philosophy, that is, they employ a divide and conquer strategy to isolate the artifacts as much as possible, both in time-frequency domain through the wavelets transform decomposition and by analyzing the independent components sources. Based on the assumption that artifactual and EEG signals are linearly mixed but statistically independent, and that propagation delays through the mixing medium are negligible, ICA seems to be an optimal tool for decomposing an identifying the source of artifactual signals effectively. In order to properly take into account either sub-Gaussian and super-Gaussian signals, the Extended-Infomax ICA algorithm (Bell and Sejnowski, 1995) is used in both approaches, which allows the computation of the unmixing matrix, so that the components are as independent as possible.

Spurious isolated oscillations are then automatically detected by a means of entropy and Kurtosis measurements. On one hand, the entropy value for EOG artifacts is expected to be low due to the regular shape so they are more predictable in comparison to neural oscillations. On the other hand, peak distributions with highly positive Kurtosis values are expected for the same type of artifacts (Mammone and Morabito, 2014). Both approaches have been compared using an analysis over all the frequency range bands (delta, theta, alpha, beta, gamma) as well as over the delta band only.

#### 2.2.1. ICA-W

EEG signals are decomposed in a series of independent components (ICs), where is expected that independent sources are separated from each other. Artifactual ICs are identified by analyzing the statistical properties in terms of Kurtosis and Multi-Scale Sample Entropy measurements. To remove as little information as possible, ICs identified as artifactual are further selected for bandpass decomposition with wavelet analysis. Decomposed wavelet independent components (WICs) require a second identification stage with the aim of zeroing only those wavelet components carrying artifactual information. Finally, the original signals are reconstructed with the inverse transforms of wavelets and ICA decompositions (Mahajan and Morshed, 2014).

#### 2.2.2. EAWICA

The original EAWICA method proposes the isolation approach of the artifactual signal component by first computing the wavelet components over the EEG signals within the frequency ranges associated with the emotion estimation task. Thus, once the information is bandpass filtered, ICA decomposition is applied to isolate artifactual data in a series of WICS. In order to automatically detect artifactual WICS, Kurtosis, and Renyi entropy measurements are used. Those marked as artifactual are further split into a series of time windows with a temporal interval of one second defined as epochs, which in case of being marked as artifactual, are zeroed to remove as little information as possible. Finally, ICA reconstruction followed by wavelet components addition is performed to reconstruct the original signals (Mammone and Morabito, 2014).

#### 2.2.3. Differences and Modifications

One of the main differences between both algorithms is that EAWICA methods improve the localization of the artifactual data by first band-passing the signals, which also helps the ICA algorithms to properly identify the sources as it takes the advantage of the redundancy by having more data. Another key difference is the way both methods apply the threshold steps to identify the artifactual data. While the ICA-W performs an automatic threshold method that works in the frequency domain (wavelet components), the EAWICA method performs in the time domain. Regarding the EAWICA threshold was restrictive, in order to improve upon this, a design decision has been taken to allow the variation of the thresholds by manually adjusting them in terms of the quartiles over the distribution values.

#### 2.2.4. Metrics

To properly compare both methods, EEG signals have been artificially contaminated with a set of artificially generated artifacts as is done by Mammone and Morabito (2014). A series of measurements are computed to compare both methods: root-mean-square error (RMSE), correlation (CORR), mutual information (MI), and coherence (C) together with timing measurements, will allow the best method to be chosen.

### 2.3. Emotion Estimation Methodology

The proposed methodology has been designed for its future use on subject dependent paradigms in the domain of HRI. This implies that the time consumption of each of the following processes must accomplish real-time constraints. Therefore, this philosophy guides the decision-making process taking into account an optimal balance between fast computation and accuracy.

#### 2.3.1. Preprocessing

EEG signals are arranged in a three-dimensional matrix containing *n* trials, *c* channels, and *s* samples at a sampling frequency, *f*_*s*_. First, given that each signal has its own scaling factor values, signals are standardized using the z-score method. Second, a filter bank, based on sixth-order Butterworth filters, is applied for all *n*, *c*, and *s*, within a set of 5 non-overlapping bandwidths: 1–4, 4–8, 8–16, 16–30, and 30–50 Hz.

#### 2.3.2. Feature Extraction Methodology

Once the data-set has been preprocessed, a set of features are computed based on the oscillatory properties of brain signals:

- Differential Entropy (DE): computed as a metric for measuring the predictability of signal *X*, whose values have a probability density function similar to a Gaussian distribution, N(μ, σ^2^), as is the case for EEG signals. It can be defined as $h\left(X\right)=\frac{1}{2}\log\left(2\pi e\sigma^{2}\right)$.
- Amplitude Envelope (AE): computed using the Hilbert transform (Boashash, 1992).
- Petrosian Fractal Dimension (PFD): defined as *PFD* = log(*N*)/(log(*N*) + log(*N*/(*N* + 0.4*N*_δ_))), where *N* is the series length, and *N*_δ_ is the number of sign changes in the signal derivative (Petrosian, 1995).
- Higuchi Fractal Dimension (HFD): Higuchi's algorithm can be used to quantify the complexity and self-similarity of a signal (Accardo et al., 1997).
- Fisher Information (FI): Fisher information is a way of measuring the amount of information that an observable random variable *X* carries about an unknown parameter θ of a distribution that models *X* (Fisher, 1925).

All features have been computed using a sliding window of 6 seconds as suggested by Candra et al. (2015), without overlapping. Each training sample represents the computed features for each time window. Features are computed for each band/channel and later concatenated for each training sample. Thus, resulting in a feature set of 435 samples with 200 features. AE has been computed with the Neuro Digital Signal Processing Toolbox (NeuroDSP) python library (Cole et al., 2019) developed at Voytek's Lab. PFD, HFD, and FI have been computed with the PyEEG python library (Bao et al., 2011).

#### 2.3.3. Feature Smoothing

Emotions are often considered static in the field of EEG to simplify the data processing for the classifiers, albeit continuous and subtle changes should be considered in the time domain. It has been noticed previously (Zheng and Lu, 2015; Zheng et al., 2015, 2017), that considering the temporal dependence and variation of emotions during the stimuli improves the performance of the training step. To do that, smoothing the feature space allows us to filter out those components that are unrelated to emotional states, thus becoming a key step for the design of an optimal methodology. In other words, smoothing the feature space deals with the amount of variability that emerges due to subtle changes in emotional states across trials and with the lack of stability over time, of the computed features.

Savitzky-Golay (SG) filtering method is proposed as an alternative to Linear Dynamic Systems (LDS). Both approaches have the property of outperforming the classification accuracy reports above the results obtained without smoothing the feature space, but also SG smoothing is significantly faster than LDS and even improves the accuracy reports.

#### 2.3.4. Feature Scaling

Feature scaling is a key step in preprocessing data. Outliers can severely damage the performance of the classifiers while looking for statistical differences. Moreover, some machine-learning algorithms for the dimensionality reduction and classification processes require data to have a predefined range of values. The process of scaling data must be performed taken into account both constraints to properly feed the algorithms in the next steps. In this paper, the Quantile-Transform method (histogram equalization to uniform distribution) followed by the Min/Max scaling method is performed. The former is a non-linear method for scaling data distributions which is robust to outliers. The later allows re-scaling in a positive range of values [0−1] as the dimensional reduction proposed method requires positive values as input.

#### 2.3.5. Dimensionality Reduction

As mentioned in the introduction, wrapper and embedded methods combine feature selection and the learning process by the use of nested cross-validation schemes but this leads to biased results when taking into account the particularities of EEG time series. Therefore, χ^2^ feature selection technique has been chosen as it is a filter-based method where the selection of features is based on the chi-squared statistic which measures the lack of independence between a feature and the target, without involving any cross-validation biased scheme nor combining the selection and the learning process.

#### 2.3.6. Set of Classifiers

Classification process has been performed using a set of eight classifiers: K-nearest neighbors, Support Vector Machine with linear and radial basis function kernels, Decision Trees, Random Forests, Ada-Boost, Gaussian Naive-Bayes, and Quadratic Discriminant Analysis. Results have been obtained with default hyper-parameter values. The Scikit-learn python library (Pedregosa et al., 2011) has been used.

#### 2.3.7. Performance Evaluation

The crucial point is to ensure that samples in the validation set are reasonably independent of the samples in the training set. Therefore, three different validation methods are reported in this paper.

- Validation across trials: Using the first nine trials as the training set and remaining six ones as the testing set for each subject and session.
- Validation across sessions: Train and test are performed over the whole set of sessions pairs for each subject.
- Validation across subjects: Leave-one-subject-out validation scheme is used.

## 3. Results

### 3.1. Online Performance Over Artificial and Real Artifactual Data

**Figure 1:**
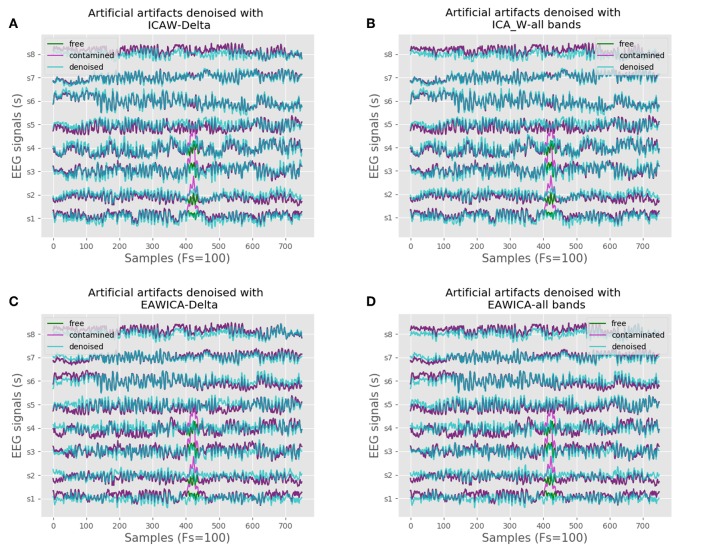
EAWICA and ICA-W methods performance comparison over artificial artifactual data. **(A)** ICA-W using only the delta band component of artifactual signals. **(B)** ICA-W using all bands components of artifactual signals. **(C)** EAWICA using only the delta band component of artifactual signals. **(D)** EAWICA using all bands components of artifactual signals.

Figure 1 shows the performance of both EAWICA and ICA-W methods applied over artifactual data. Both methods have been applied, on one hand, using all the frequency ranges of interest (delta, theta, alpha, beta, gamma) and, on the other hand, over the slowest frequency range (delta). It can be noted that ICA-W focuses on the artifactual data better than EAWICA, moreover, the latter seems to affect the signal in all the frequency ranges. As a comparison, a set of metrics are shown in Table 1. Taking into account these metrics, ICA-W outperforms the obtained results in terms of CORR, MI, and RMSE, with regard to the artifactual-free signals. Concerning time consumption, EAWICA performs the filtering process in 0.19 s while still having low RMSE and high CORR and MI results, and ICA-W takes 0.6 s.

**Table 1:** Metrics comparison for the four cases.

	**RMSE**	**Correlation**	**Mutual information**	**Time (s)**
EAWICA - ALL	0.27	0.87	0.66	0.19
ICA-W - ALL	0.16	0.92	0.87	0.60
EAWICA - DELTA	0.29	0.86	0.64	0.36
ICA-W DELTA	0.14	0.93	0.93	0.68

**Figure 2:**
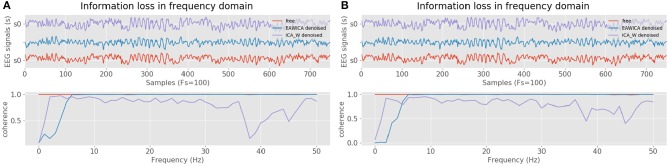
Coherence estimates between filtered (EAWICA and ICA-W) signal and artifact-free signal. **(A)** Coherence estimation after artifact removal using all frequency bands. **(B)** Coherence estimation after artifact removal using only the delta band.

Signal filtering performance can also be evaluated in terms of the coherence lost relative to the original artifact-free signal. Coherence is referred to as the cross-frequency spectrum of two signals, it is a measurement of the loss of the filtered signals relative to the original, in the frequency domain. Figure 2A shows the coherence estimates between both the EAWICA and ICA-W filtering methods relative to the artifact-free signals, where it can be noted that EAWICA applied over all frequency ranges, severely damages the original signal in all the frequency spectrum. On the contrary, EAWICA applied over the delta band outperforms the signal cross-frequency measurements against ICA-W as it can be shown in Figure 2B.

**Figure 3:**
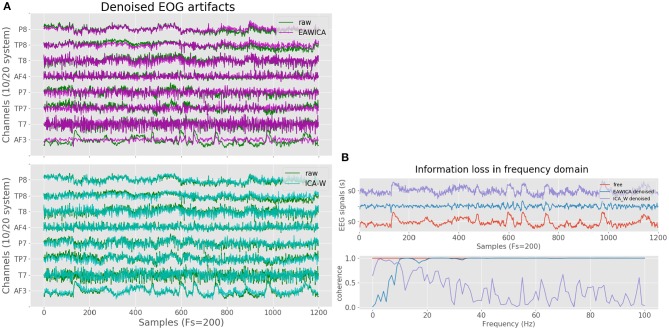
EAWICA and ICA-W methods performance comparison over real EEG data. **(A)** Comparison between EAWICA and ICA-W artifact removal strategies in the time domain. **(B)** Coherence comparison between EAWICA and ICA-W artifact removal strategies in the frequency domain.

Artificial artifactual data is useful in order to compare the performance of a set of algorithms taking into account the basic truth, but real domains often involve more complex signals. Therefore, both methods need to be compared against real artifactual EEG data, which in this case is obtained from the SEED database. Figure 3A shows the performance of both algorithms and for the real case EAWICA seems to outperform ICA-W. Figure 3B better shows how the EOG artifacts are removed by EAWICA while still maintaining all the information in the frequency spectrum. Regarding time consumption, EAWICA required 0.4 s and ICA-W 2.2 s both for filtering 6 s of 8 signals with a sampling rate of 200 Hz. Taking all these results into account, EAWICA has been selected for the methodology.

### 3.2. Emotion Estimation

In machine learning applied to emotion estimation, the standard K-fold cross-validation is often applied. At that point, there is a key issue that arises when the performance of the models is evaluated. These methods cannot be directly used with time series data as they assume that there is no relationship between the observations, that is, each observation must be independent while in fact, they are not. The EEG time series data in emotion estimation strongly correlated along the time axis. The randomization performed with cross-validation methods make it likely that for each sample in the validation set, numerous strongly correlated samples exist in the training set but this defeats the very purpose of having a validation set: the model has prior information about the validation set, leading to optimistic performance reports on it. Such an analysis could provide an insight into how the selected model works, or if there exists a statistical difference between samples, but not if a correlation between these statistical differences and the task at hand is really present. Furthermore, any estimate of the performance will be optimistic and any conclusion based on this performance will be biased and could be completely wrong. This problem is not only related to the classification step, as a result, it also arises in the dimensionality reduction step if predefined algorithms are used, which do not take into account these assumptions.

Therefore, to properly evaluate the performance, three different validation schemes were used. The evaluation has been performed taking into account different subsets of features, ranging from 1 to 200.

#### 3.2.1. Feature Smoothing

**Figure 4:**
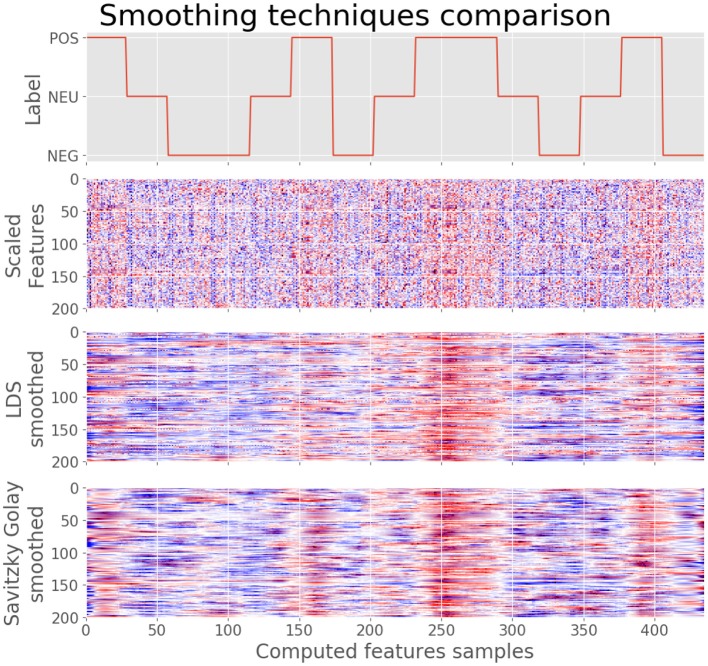
Feature smoothing applied over the feature space. At the **(top)**, the label of each short film in the time domain. Below, the feature space after Quantile-Transform normalization. Then, feature space smoothed using LDS. At the **(bottom)**, feature space smoothed using Savitzky-Golay filtering. As it can be noted, label smoothing clarifies the correlation of the feature space regarding labels.

Figure 4 shows the comparison of applying LDS or SG methods on the feature space. It can be clearly noted that smoothing the features space makes it possible to clearly observe the correlation with the corresponding targets. Moreover, the SG method makes the features space even less noisy. Therefore, although the LDS smoothing method works well eliminating the variability, the SG method outperforms the obtained results, both in terms of removing such variability, as well as with regard to the time consumption needed. While LDS needs roughly 200 s for smoothing a feature space of 435 samples × 200 features, SG takes approximately 73 ms.

#### 3.2.2. Trial Validation Tests

**Figure 5:**
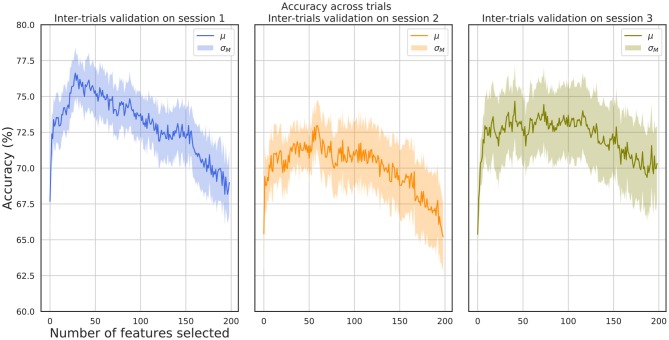
Inter-trial validation accuracy. The figure on the left shows the accuracy results for validation between trials in session 1, the middle figure for session 2 and the right figure for session 3.

Figure 5 shows the μ ± σ_*M*_ performance for inter-trial validation tests for all subjects in each session. A set of 9 trials for each subject have been selected for training while 6 are used as a test set, were 2 trials are present for each class (positive, negative, neutral). For each training step, a set of features ranging from 1 to 200 have been used. This evaluation provides an insight into the robustness of the method in terms of generalization performance of the model in a more realistic scenario for the unseen. The best mean accuracy report for the best subset of features for each subject is (82.3 ± 4.4) % for session 1, (78.9 ± 5.7)% for session 2, and (80.5 ± 8.6)% for session 3.

#### 3.2.3. Sessions Validation Tests

**Figure 6:**
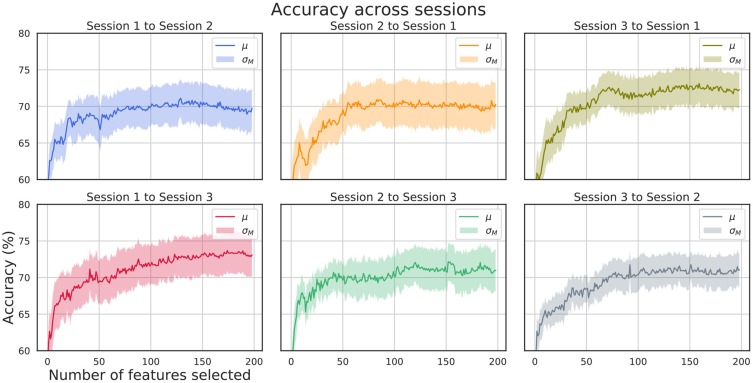
Inter-session validation accuracy. Six different combinations of inter-session validation are shown.

Figure 6 shows the μ ± σ_*M*_ performance for inter-session validation tests for all subjects and for all session to session pairs. For each training step, a set of features ranging from 1 to 200 have been used. As the SEED database consists of the same set of stimuli for each session, these results prove the stability of the selected features over time. The best mean accuracy report for the best subset of features for each subject is (74.6 ± 8.8)% for session 1 to session 2, (74.9 ± 11.1)% for session 2 to session 1, (76.8 ± 8.1)% for session 3 to session 1, (77.0 ± 10.1)% for session 1 to session 3, (76.4 ± 8.4)% for session 2 to session 3, and (75.9 ± 6.7)% for session 3 to session 2.

#### 3.2.4. One Subject Out Validation Tests

**Figure 7:**
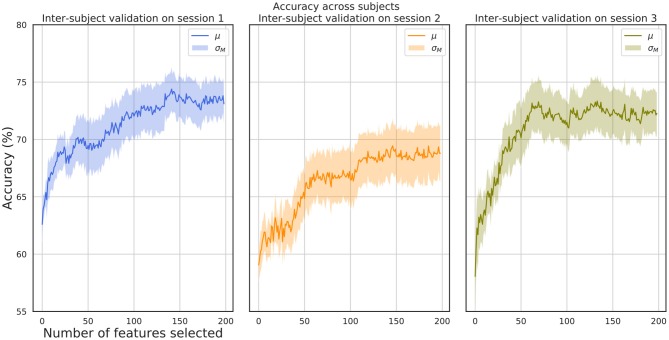
One subject out validation accuracy with respect to selected features. Inter-subject validation for the three sessions.

Figure 7 shows the μ ± σ_*M*_ performance for inter-subject validation tests. A leave-one-out subject evaluation scheme has been used. For each training step, a set of features ranging from 1 to 200 have been used. This validation scheme provides an insight into the robustness of the selected set of features for the subject independent paradigm, confirming that underlying common processes exist across subjects and that the selected features are closely related to invariant properties of the brain oscillations dynamics for the evoked emotion experimentation. The best mean accuracy report for the best subset of features for each subject is (77.6 ± 5.6)% for session 1, (73.4 ± 6.7)% for session 2, and (77.1 ± 7.7)% for session 3.

#### 3.2.5. Time Consumption

The main objective of this methodology is to perform under real-time constraints while having high accuracy results in terms of emotion estimation. Thus, time consumption analysis is needed as a comparison for the design decisions. Methodologies based on real-time constraints have to deal with the following key processing steps:

- Time consumption of the artifact filtering process and feature extraction steps.
- Time consumption of the feature smoothing and scaling steps.
- Time consumption of the classifying and fine-tuning step.

Feature smoothing has shown to be a key step to further improve the performance of models but those filtering processes cannot be performed over unique samples. Therefore, a set of samples for each trial should be present in the training set before smoothing the features. Thus, the experimental paradigm in a real scenario needs to be performed on three main stages. First, a real-time signal acquisition while the subject is stimuli evoked. In that stage, signals could be stored without any filtering process to alleviate the computation effort of the acquisition application. The second should consist of the model training step. Each EEG set of signals would be split on windowed samples for the processing of training sets. This stage should perform the following steps: artifact removal, feature extraction, feature smoothing, and scaling, and finally model training. For this stage, it is a requirement for the time consumption to be short, in order to reduce the amount of time the subject under study is waiting. Finally, the following steps for each acquired sample should be performed: online artifact removal, feature extraction, smoothing, and scaling taking into account training samples to properly transform the computed features, and the final prediction step.

For this experimental paradigm, several time metrics have been computed for each step. As a comparison, Table 2 shows the details of each processing step for the case of offline training and online prediction stages. The software used was the Scikit-learn python library (Pedregosa et al., 2011) running in an Intel Core i7-8700 K (3.70 GHz).

**Table 2:** Time consumption regarding offline training and online prediction stages.

	**Offline training**	**Online prediction**
(Samples, features)	(435, 200)	(1, 200)
Artifact removal	151.89(23)s	200(16)ms
Feature extraction	79.27(3)s	182.25(8)ms
LDS smoothing	149.35(7)s	None
SG smoothing	67.31(1)ms	67.31(1)ms
Normalize	98.42(1)ms	98.42(1)ms
Feature selection	1.87(5)ms	None
Training/Predicting	873.65(62)ms	38.8(11)μ*s*
Total	381.55s	548.02ms

Offline training is analyzed taking into account one subject session, which means using 435*200(*samples*/*features*). The process of online filtering performed in (151.89 ± 0.23) s. The feature extraction step required (79.27 ± 0.03) s. Concerning feature smoothing, LDS required (149.35 ± 0.07) s, while SG was able to perform the same task in approximately (67.31 ± 0.01) ms. The scaling process was performed in approximately (98.42 ± 0.01) ms. Feature selection was computed for the worst-case scenario, selecting only the best feature, which required (1.87 ± 0.05) ms. The classifying step was proposed for a set of eight classifiers. The proposed approach consists of selecting a range of features taking into account the aforementioned results to reduce the amount of time to find the number of features that best generalizes over the unseen. The amount of time needed for each classification of a number X of selected features for the set of eight classifiers is approximately (873.65 ± 0.62) ms. Furthermore, there is no need for fine-tuning, as the previous results show that the methodology is robust enough without hyper-parameter tuning.

On the other hand, the online prediction is analyzed regarding the time consumption for one unique sample. Online artifact removal is carried out in (200 ± 16) ms, while feature extraction is performed in (182.25 ± 0.08) ms. Based on previous results, SG smoothing is used instead of LDS, as the processing time has been shown to reduce. Furthermore, feature selection is not computed as the set of best features are previously determined during the offline training stage. As SG smoothing and normalization steps cannot be performed over one unique sample, the strategy must involve the set of features computed for the offline training stage, thus, the time required is the same as in the offline training. Stacking the incoming samples in this data structure allows for both processes to be applied, and once the incoming sample has been smoothed and normalized, taking into account previous training samples, it is unstacked for the final prediction step over the best selected model during the offline training, at the time cost of (38.8 ± 1.1)μs.

## 4. Discussion

The proposed methodology accomplishes the main processing steps required for a real-time emotion estimation approach and overcomes the many difficulties presented in this field of study.

Artifact removal is the first step of the whole process and therefore plays a very important role in the outcome. As mentioned earlier, only two methods for online artifact removal were tested, EAWICA and ICA-W, since they show to be feasible for real-time constraints. EAWICA outperforms ICA-W when using real EEG data and was therefore chosen as part of this method. A modified version of EAWICA, constrained to the delta band, was used to reduce artifacts (EOG), and therefore reduce computation time.

In this paper, 8 electrodes were used, six temporal electrodes and two prefrontal, placed at AF3, T7, TP7, P7, AF4, T8, TP8, and P8, since these were previously shown in the literature to be the best brain locations to use for emotion estimation (Zheng and Lu, 2015; Zheng et al., 2017). The chosen set of electrodes showed to be a good choice since it offered a proper balance between the more informative electrodes and redundancy for the classifying step (Kohavi and John, 1997), and achieved good results. Due to the great number of electrodes employed, high dimensional space is often a limitation in the domain of EEG signal analysis. Such signals have a complicated structure since their intrinsic properties are non-linear and non-stationary, thus, the need for balance between a wide dimensional space of features that must be treated carefully with feature reduction techniques, often biased by the statistics in hand (Fan and Li, 2006), and a set of low components feature space, which would be desirable.

The robustness of the methodology is higher when only a subset of eight temporal and prefrontal electrodes is used, leading to a feature space with fewer dimensions. On the other hand, the results show that in general, a subset ranging from 50 to 100 features leads to the optimal accuracy results, probably because the redundancy on the features space enhances the performance of the classifiers.

In the HRI domain, it is crucial to ensure that real-time emotion estimation is a quick and versatile process. The set of selected features chosen for this methodology is easy to compute in any type of computer and can be easily implemented in any programming language, allowing the quick development of portable systems with high accuracy results, as is the case for the openBCI system. Also, these features allow the interpretation of the phenomenon under study, as they are direct measurements of the properties of brain patterns, being far from black-box techniques, which use deep-learning approaches such as auto-encoders (Chai et al., 2016), or very complex features with difficult interpretation in biological terms (Zheng et al., 2015).

A proper validation scheme is important for every novel methodology for it to be comparable to those in literature. As mentioned earlier, predefined cross-validation schemes for supervised learning algorithms are not suitable for model performance evaluations in EEG emotion estimation (Tashman, 2000). Liu et al. (2017) proposed a real-time methodology which recognized eight different brain affective states with a three-step level classification. This methodology is therefore comparable to the proposed method, however, the validation scheme which was used is not clearly stated, and could, therefore, be backtesting, IT or cross-validation. Without this knowledge, a proper comparison of the results can not be achieved. On the other hand, since different validation schemes were found to be used in state of the art, this method was validated using IT, IS, and LOO (see Table 3), in order to properly compare the results. As observed in Table 3, the proposed method is close to the results obtained by previous studies in terms of accuracy for the cases of IT and IS schemes, and outperforms the results obtained by previous studies in the LOO scheme, which is the most complex due to inter-subject variability.

**Table 3:** State of the art comparison.

	**Validation scheme**	**Database: Emotional model**	**Accuracy (%)**
Khosrowabadi et al. (2014)	Cross-validation	DEAP: valence/arousal	70.83/71.43
Zheng and Lu (2015)	IT	SEED: [pos., neg., neu.]	86.65
Zheng et al. (2017)	LOO and IS	SEED: [pos., neg., neu.]	60.93 and 79.28
Tripathi et al. (2017)	LOO	DEAP: valence/arousal	66.79/57.58
Song et al. (2018)	IT	SEED: [pos., neg., neu.]	90.4
Liu et al. (2017)	Not specified	Own produced: [neu., non-neu.]/[pos., neg.]/[joy, amusement, tenderness]/ [sad, angry, fear, disgust]	92.26/86.63/86.43/65.09
Proposed method	IT/IS/LOO	SEED: [pos., neg., neu.]	82.27/76.36/77.59

## 5. Conclusion

Our method has proved to be robust and fast, reaching comparable results to state of the art in subject dependent and independent analysis for EEG emotion recognition. An accurate and computationally light EEG emotional estimation methodology could allow the use of portable and cheap devices in the domain of emotional HRI.

This method uses a three-categories emotional model; however, for more complex emotional models, more complex deep learning strategies must be implemented (Zheng et al., 2018; Zhao et al., 2019). Therefore, even maintaining the three-categories emotional model, this method could be improved by altering the filtering methods and with a better coding strategy, that is, with a set of features that better describe the invariant relationships of emotionally evoked brain patterns and their corresponding categories.

Since this method has proven to be fast, over 1 s total processing time, and reliable, 82.27, 76.36, 77.59% for {IT, IS, LOO} validation schemes respectively, it fulfills the proposed task. Therefore, it is an optimal methodology for HRI, that could further the research in this field.

## Data Availability Statement

Publicly available datasets were analyzed in this study. This data can be found here: http://bcmi.sjtu.edu.cn/~seed/downloads.html.

## Author Contributions

MV-C and JÁ-S developed the methodology. JF-V and EF designed and supervised the theoretical approach and results.

### Conflict of Interest

The authors declare that the research was conducted in the absence of any commercial or financial relationships that could be construed as a potential conflict of interest.

## References

[B1] Accardo A. Affinito M. Carrozzi M. Bouquet F. (1997). Use of the fractal dimension for the analysis of electroencephalographic time series. Biol. Cybern. 77, 339–350. 10.1007/s004220050394 9418215

[B2] Bao F. S. Liu X. Zhang C. (2011). Pyeeg: an open source python module for eeg/meg feature extraction. Comput. Intell. Neurosci. 2011:406391. 10.1155/2011/406391 21512582 PMC3070217

[B3] Bell A. J. Sejnowski T. J. (1995). An information-maximization approach to blind separation and blind deconvolution. Neural Comput. 7, 1129–1159. 10.1162/neco.1995.7.6.1129 7584893

[B4] Boashash B. (1992). Estimating and interpreting the instantaneous frequency of a signal. I. Fundamentals. Proc. IEEE 80, 520–538. 10.1109/5.135376

[B5] Candra H. Yuwono M. Chai R. Handojoseno A. Elamvazuthi I. Nguyen H. T.. (2015). Investigation of window size in classification of EEG-emotion signal with wavelet entropy and support vector machine, in Engineering in Medicine and Biology Society (EMBC), 2015 37th Annual International Conference of the IEEE (IEEE), 7250–7253. 10.1109/EMBC.2015.7320065 26737965

[B6] Chai X. Wang Q. Zhao Y. Liu X. Bai O. Li Y. (2016). Unsupervised domain adaptation techniques based on auto-encoder for non-stationary EEG-based emotion recognition. Comput. Biol. Med. 79, 205–214. 10.1016/j.compbiomed.2016.10.019 27810626

[B7] Cole S. Donoghue T. Gao R. Voytek B. (2019). Neurodsp: a package for neural digital signal processing. J. Open Source Softw. 4:1272 10.21105/joss.01272

[B8] Davidson R. J. (1992). Anterior cerebral asymmetry and the nature of emotion. Brain Cogn. 20, 125-151. 10.1016/0278-2626(92)90065-T 1389117

[B9] Davidson R. J. Fox N. A. (1982). Asymmetrical brain activity discriminates between positive and negative affective stimuli in human infants. Science 218, 1235–1237. 10.1126/science.7146906 7146906

[B10] Fan J. Li R. (2006). Statistical challenges with high dimensionality: feature selection in knowledge discovery. arXiv preprint math/0602133.

[B11] Fisher R. A. (1925). Theory of statistical estimation, in Mathematical Proceedings of the Cambridge Philosophical Society, Vol. 22 (Cambridge: Cambridge University Press), 700–725.

[B12] Khosrowabadi R. Quek C. Ang K. K. Wahab A. (2014). ERNN: a biologically inspired feedforward neural network to discriminate emotion from EEG signal. IEEE Trans. Neural Netw. Learn. Syst. 25, 609–620. 10.1109/TNNLS.2013.2280271 24807454

[B13] Kohavi R. John G. H. (1997). Wrappers for feature subset selection. Artif. Intell. 97, 273–324. 10.1016/S0004-3702(97)00043-X

[B14] Kragel P. A. LaBar K. S. (2016). Decoding the nature of emotion in the brain. Trends Cogn. Sci. 20, 444–455. 10.1016/j.tics.2016.03.011 27133227 PMC4875847

[B15] Liu Y. Sourina O. Nguyen M. K. (2010). Real-time EEG-based human emotion recognition and visualization, in 2010 International Conference on Cyberworlds (Singapore: IEEE), 262–269.

[B16] Liu Y.-J. Yu M. Zhao G. Song J. Ge Y. Shi Y. (2017). Real-time movie-induced discrete emotion recognition from EEG signals. IEEE Trans. Affect. Comput. 9, 550–562. 10.1109/TAFFC.2017.2660485

[B17] Mahajan R. Morshed B. I. (2014). Unsupervised eye blink artifact denoising of EEG data with modified multiscale sample entropy, kurtosis, and wavelet-ica. IEEE J. Biomed. Health Inform. 19, 158–165. 10.1109/JBHI.2014.2333010 24968340

[B18] Mammone N. Morabito F. (2014). Enhanced automatic wavelet independent component analysis for electroencephalographic artifact removal. Entropy 16, 6553–6572. 10.3390/e16126553

[B19] Muthukumaraswamy S. (2013). High-frequency brain activity and muscle artifacts in MEG/EEG: a review and recommendations. Front. Hum. Neurosci. 7:138. 10.3389/fnhum.2013.00138 23596409 PMC3625857

[B20] Pedregosa F. Varoquaux G. Gramfort A. Michel V. Thirion B. Grisel O. (2011). Scikit-learn: machine learning in Python. J. Mach. Learn. Res. 12, 2825–2830. Available online at: https://arxiv.org/abs/1201.0490

[B21] Petrosian A. (1995). Kolmogorov complexity of finite sequences and recognition of different preictal EEG patterns, in Proceedings Eighth IEEE Symposium on Computer-Based Medical Systems (Texas: IEEE), 212–217.

[B22] Rani M. S. B. A. Mansor W. (2009). Detection of eye blinks from EEG signals for home lighting system activation, in 2009 6th International Symposium on Mechatronics and Its Applications, ISMA 2009 (Sharjah), 5164828.

[B23] Roseman I. J. Spindel M. S. Jose P. E. (1990). Appraisals of emotion-eliciting events: testing a theory of discrete emotions. J. Pers. Soc. Psychol. 59, 899–915. 10.1037/0022-3514.59.5.899

[B24] Russell J. A. (1980). A circumplex model of affect. J. Pers. Soc. Psychol. 39, 1161–1178. 10.1037/h0077714

[B25] Song T. Zheng W. Song P. Cui Z. (2018). EEG emotion recognition using dynamical graph convolutional neural networks. IEEE Trans. Affect. Comput. 1–1. 10.1109/TAFFC.2018.2817622

[B26] Tashman L. J. (2000). Out-of-sample tests of forecasting accuracy: an analysis and review. Int. J. Forecast. 16, 437–450. 10.1016/S0169-2070(00)00065-0

[B27] Tripathi S. Acharya S. Sharma R. D. Mittal S. Bhattacharya S. (2017). Using deep and convolutional neural networks for accurate emotion classification on deap dataset, in AAAI (New York, NY), 4746–4752.

[B28] Yin Z. Zhao M. Wang Y. Yang J. Zhang J. (2017). Recognition of emotions using multimodal physiological signals and an ensemble deep learning model. Comput. Methods Prog. Biomed. 140, 93–110. 10.1016/j.cmpb.2016.12.005 28254094

[B29] Zhao L.-M. Li R. Zheng W.-L. Lu B.-L. (2019). Classification of five emotions from EEG and eye movement signals: complementary representation properties, in 2019 9th International IEEE/EMBS Conference on Neural Engineering (NER) (Shanghai: IEEE), 611–614. 10.1109/EMBC.2019.8857476 31947389

[B30] Zheng W.-L. Liu W. Lu Y. Lu B.-L. Cichocki A. (2018). Emotionmeter: a multimodal framework for recognizing human emotions. IEEE Trans. Cybern. 49, 1110–1122. 10.1109/TCYB.2018.2797176 29994384

[B31] Zheng W.-L. Lu B.-L. (2015). Investigating critical frequency bands and channels for EEG-based emotion recognition with deep neural networks. IEEE Trans. Auton. Mental Dev. 7, 162–175. 10.1109/TAMD.2015.2431497

[B32] Zheng W.-L. Zhang Y.-Q. Zhu J.-Y. Lu B.-L. (2015). Transfer components between subjects for EEG-based emotion recognition, in 2015 International Conference on Affective Computing and Intelligent Interaction (ACII) (IEEE), 917–922.

[B33] Zheng W.-L. Zhu J.-Y. Lu B.-L. (2017). Identifying stable patterns over time for emotion recognition from EEG. IEEE Trans. Affect. Comput. 10, 417–429. 10.1109/TAFFC.2017.2712143

